# The influence of varied parenteral nutrition protocols on the surgical incidence and prognosis of retinopathy of prematurity in preterm neonates

**DOI:** 10.3389/fped.2025.1629765

**Published:** 2025-08-06

**Authors:** Yafei Sun, Teng Wang, Ruixia Lin, Xuejun Xu

**Affiliations:** ^1^ Department of Ophthalmology, Qingdao Chengyang District People’s Hospital, Qingdao, Shandong, China; ^2^ Department of Pediatrics, Qingdao Chengyang District People’s Hospital, Qingdao, Shandong, China; ^3^ Department of Ophthalmology, The Fifth People’s Hospital of Qingdao, Qingdao, Shandong, China

**Keywords:** preterm infants, n-3 fatty acid-enriched lipid emulsions, medium/long-chain lipid emulsions, retinopathy of prematurity, parenteral nutrition

## Abstract

**Objective:**

This retrospective cohort study aimed to assess the impact of different parenteral nutrition (PN) protocols on the incidence and prognosis of retinopathy of prematurity (ROP) in preterm infants.

**Methods:**

Medical records of 87 preterm infants admitted to the neonatal intensive care unit between October 2019 and October 2022 were retrospectively analyzed. The infants were non-randomly allocated into two groups based on the PN protocols they received: the fish oil group (41 cases) received PN with high n-3 fatty acid-containing lipid emulsions, while the non-fish oil group (46 cases) received PN with medium and long-chain lipid emulsions. Fatty acid profiles were assessed on the first day of hospitalization and after 14 days of PN. The incidence of ROP at 4 and 6 weeks after birth was compared, along with the time taken to regain birth weight, achieve full enteral feeding, duration of mechanical ventilation, and ROP surgical rate during hospitalization.

**Results:**

On the first day of hospitalization, there were no significant differences in DHA, EPA, and AA levels between the two groups. However, after 14 days of PN, the fish oil group showed significantly higher DHA levels and lower AA levels compared to the non-fish oil group. The fish oil group required less time to achieve full enteral feeding compared to the non-fish oil group. There were no significant differences in other blood parameters between the two groups. The levels of liver enzymes (ALT, TBA, AST, γ-GT) were significantly lower in the fish oil group. There were no significant differences in the overall incidence of ROP and mild ROP between the two groups at 4 and 6 weeks after admission. However, the fish oil group had a significantly higher incidence of severe ROP and a significantly lower surgical rate compared to the non-fish oil group.

**Conclusion:**

Early administration of lipid emulsions enriched with n-3 fatty acids in preterm infants has a preventive effect on severe ROP. This intervention is associated with higher serum DHA levels and lower AA levels, shorter time to achieve full enteral feeding, and reduced surgical rate for ROP. Further research is needed to optimize PN strategies in preterm infants with ROP.

## 1 Introduction

Retinopathy of prematurity (ROP) is a potentially blinding condition that affects preterm neonates, especially those with very low birth weights or born extremely prematurely. ROP is characterized by abnormal growth of retinal blood vessels, which can lead to retinal detachment and irreversible vision loss if left untreated (1, 2). Despite advancements in neonatal care, ROP remains a significant concern in neonatal intensive care units worldwide. According to recent statistics, approximately 1.26 million children worldwide suffer from blindness, with ROP being the leading preventable cause of childhood blindness (3). Untreated advanced ROP (Stages 4–5) may cause cataracts through inflammatory mediators and lens metabolic disruption secondary to retinal detachment, alongside secondary glaucoma, collectively leading to permanent vision loss (4, 5). Moreover, late-stage treatment of ROP is challenging and costly, underscoring the importance of early prevention and management.

Premature infants often face challenges due to their immature gastrointestinal function and underdeveloped organs, making it difficult for them to tolerate enteral feeding in the early stages of life. Additionally, they are prone to underlying diseases and serious complications, including malnutrition and infections (6). Consequently, many premature infants require parenteral nutrition (PN) (7). Lipid emulsions play an essential role in PN as they not only provide a significant source of energy but also supply essential fatty acids (8). One important fatty acid is docosahexaenoic acid (DHA), belonging to the n-3 fatty acid family. DHA is synthesized from alpha-linolenic acid and arachidonic acid (AA). It is a critical component of retinal cell membranes and crucial for neurodevelopment (9). Compared to full-term infants, premature infants have lower serum levels of DHA due to limited lipid stores and require higher daily DHA intake. Furthermore, premature infants have a weaker capacity to synthesize DHA (10). Although some studies have demonstrated preventive effects of n-3 fatty acid-enriched lipid emulsions against ROP, limited clinical research has been conducted in this area.

The pathogenesis of ROP is complex and multifactorial, but emerging evidence suggests that nutritional factors may contribute to its development and progression. PN plays a crucial role in the care of preterm neonates, providing essential nutrients when enteral feeding is not possible or insufficient. However, the optimal composition of PN for these vulnerable infants is still a subject of ongoing research and debate.

Studies investigating the influence of varied PN protocols on the incidence and prognosis of ROP in preterm neonates have yielded conflicting results. Some studies suggest a potential association between specific nutrient deficiencies or imbalances and an increased risk of ROP, while others fail to establish a significant link. The heterogeneity in research findings and the lack of consensus regarding optimal nutrition regimens highlight the need for further investigation in this area.

The objective of this study is to investigate the impact of n-3 fatty acid-enriched lipid emulsions in PN for premature infants who require PN, with a control group for comparison. The study aims to further explore the influence of these lipid emulsions on retinal development in premature infants, ultimately aiming to improve their prognosis.

## 2 Methods

### 2.1 Inclusion and exclusion criteria

The inclusion criteria were as follows: (1) infants who received fish oil or soybean oil-based lipid emulsion therapy within 24 h after birth, (2) those who received PN for more than 14 days, and (3) infants with a birth weight of less than 2000g and a gestational age of less than 34 weeks. These criteria align with the Chinese guidelines for ROP screening (11), which recommend screening for infants with birth weight ≤2,000 g or GA ≤34 weeks, as well as those with additional risk factors (e.g., prolonged oxygen therapy), to account for regional variations in ROP incidence and ensure comprehensive screening. Exclusion criteria included: (1) infants with congenital severe malformations, (2) infants with congenital metabolic disorders, and (3) infants exhibiting signs or symptoms of congenital infections. This study was conducted in accordance with the ethical regulations of the Declaration of Helsinki. Qingdao Chengyang District People's Hospital agrees to conduct an ethical investigation and agrees to waive informed consent.

### 2.2 Study population

A retrospective analysis was conducted on the medical records of 87 cases of premature infants who were admitted to our neonatal intensive care unit between October 2019 and October 2022. Among them, there were 45 males and 42 females, with an average gestational age of 31.75 ± 2.04 weeks and an average birth weight of 1,592.41 ± 156.79 g. The study subjects were non-randomly allocated into two groups based on existing clinical PN regimens: a fish oil group (41 cases), receiving n-3 fatty acid-enriched lipid emulsion for PN, and a non-fish oil group (46 cases), receiving medium- and long-chain lipid emulsion for PN. Prenatal factors (maternal hypertension, chorioamnionitis, intrauterine growth restriction, antenatal corticosteroid administration) and neonatal factors (Apgar scores at 1/5 min, resuscitation type, surfactant administration, ventilation mode, Fraction of Inspired Oxygen (FiO₂), Patent Ductus Arteriosus (PDA), Necrotizing Enterocolitis (NEC), Bronchopulmonary Dysplasia (BPD), sepsis) were extracted from medical records to account for ROP confounders.

### 2.3 Standard nutritional practices

The fish oil group received parenteral nutrition using an n-3 fatty acid-enriched lipid emulsion that was rich in n-3 fatty acids. This particular lipid emulsion was manufactured by Fresenius Kabi Huaren Pharmaceuticals Co., Ltd. and had a drug registration number J20130038. Each vial contained 100 ml and included soybean oil (6 g/dl), medium-chain triglycerides (6 g/dl), olive oil (5 g/dl), and fish oil (3 g/dl). On the other hand, the non-fish oil group received a medium- and long-chain lipid emulsion via parenteral administration. This lipid emulsion was manufactured by Chongqing Pharmaceutical Co., Ltd. and had a drug registration number H20113382. Each vial contained 100 ml and contained soybean oil (5 g/dl), medium-chain triglycerides (5 g/dl), lecithin (1.2 g/dl), and glycerol (2.5 g/dl).

The parenteral nutrition support method included glucose, pediatric compound amino acids, lipid emulsion, vitamins, and electrolytes. Initially, the dose of amino acids and lipid emulsion administered was 0.5–1 g/(kg·d), gradually increased to 2.0–3.0 g/(kg·d). The initial dose of glucose was 6–8 g/(kg·d), and it was increased gradually based on the child's glucose tolerance level, not exceeding 12–16 g/(kg·d). Continuous nutrition delivery for 24 h was achieved using a peripheral or central venous microinfusion pump.

All infants received colostrum (where available) within 24 h of birth. Enteral feeding was initiated as tolerated (10–20 ml/kg/day), advanced by 10–20 ml/kg/day, with PN weaned proportionally. All infants received PN for ≥14 days; enteral feeding before day 14 supplemented but did not replace PN.

### 2.4 Observation target

Infants received non-pharmacological pain relief (pacifier dipped in 24% sucrose solution) 2 min before examinations to minimize distress. Blood samples collected during routine clinical monitoring on admission and after 14 days of lipid emulsion usage were retrospectively analyzed. Venous blood (3 ml) was drawn under aseptic conditions from the antecubital vein using a 24-gauge needle, collected in EDTA tubes, centrifuged at 3,000 rpm for 10 min, and stored at −80°C until analysis. Fatty acid profiling (DHA, EPA, AA as percentage of total fatty acids) was performed on stored RBC samples using Agilent 6890 NGC gas chromatography as part of our institutional protocol for high-risk preterm infants requiring prolonged PN support. Clinical parameters including time to regain birth weight, achieve full enteral feeding, and mechanical ventilation duration were extracted from medical records.

An automatic biochemical analyzer and an automatic blood cell analyzer were utilized to determine the blood indicators of the two groups of infants.

Changes in hepatobiliary function in the two groups were evaluated by measuring levels of alanine aminotransferase (ALT), total bile acid (TBA), aspartate aminotransferase (AST), and gamma-glutamyl transferase (γ-GT) in preterm infants.

ROP data were collected by three ophthalmologists who employed the international ROP classification. The examination was conducted using a binocular indirect ophthalmoscope (Heine Omega 500®, Germany) with a 28-diopter condensing lens. Pupillary dilation was achieved using 0.5% tropicamide eye drops (Santen Pharmaceutical Co., Japan), administered twice (5 min apart) 30 min before examination. Examinations began in the fourth week after admission and continued until retinal vascularization or a stable condition was attained. The severity of ROP lesions was classified according to the International Classification of Retinopathy of Prematurity (ICROP) (12). Stages 1–2 were categorized as mild ROP, while stages 3–5 were considered severe ROP. The occurrence of ROP during the fourth and sixth week after birth was documented for the infants.

The surgical rate of ROP, including laser therapy and vitreoretinal surgery, was compared between the two groups. Treatment followed the Early Treatment for Retinopathy of Prematurity (ETROP) guidelines (13) and institutional protocols. Laser therapy was the preferred treatment for Zone II non-posterior ROP or cases with significant membrane neovascularization. Indications and methods for vitreoretinal surgery were as follows: (1) Zone I ROP, posterior Zone II ROP, aggressive ROP with progressive proliferative membrane, and retinal hemorrhage were considered for vitreoretinal surgery. (2) Relatively aggressive vitreoretinal surgery could be considered if Stage 4a ROP displays progression and tractional retinal detachment tends to involve the macula. (3) Stage 4b ROP and Stage 5 ROP can undergo vitreoretinal surgery, and combined lens extraction surgery can be performed if necessary. The retinas were divided into three zones for classification: Zone I, a circular area centered on the optic disc with a radius twice the distance from the optic disc to the fovea; ROP in this zone is particularly severe. Zone II, the circular area within the distance from the optic disc to the nasal ora serrata, excluding Zone I. Zone III, the temporal crescent-shaped area outside Zone II, which is a high-risk area for ROP.

### 2.5 Statistical analysis

Data analysis for this study utilized SPSS 25.0 (SPSS Inc., Chicago, IL, USA) as the statistical software. The *t*-test and chi-square test were employed for analyzing different types of data. Data normality was assessed using Shapiro–Wilk tests. Normally distributed continuous variables are expressed as mean ± SD and compared via independent *t*-tests; non-normal variables were compared via Mann–Whitney U tests. Categorical variables were presented as counts (%) and analyzed by *χ*^2^ test or Fisher's exact test when expected cell counts were ≤5. The significance level for all tests was set at *α* = 0.05, and a *P*-value less than 0.05 was deemed statistically significant.

## 3 Results

### 3.1 Baseline characteristics and clinical features

There were no significant differences observed between the two groups in terms of gender (20/21 vs. 25/21, *χ*^2^ = 0.269, *P* = 0.604), gestational age (31.67 ± 2.28 vs. 31.54 ± 2.05, *t* = 0.280, *P* = 0.780), and birth weight (1,593.17 ± 159.32 vs. 1,587.24 ± 160.26, *t* = 0.173, *P* = 0.863) as shown in Table 1. Additionally, prenatal and neonatal risk factors for ROP were comparable between groups (*P* > 0.05, Tables 2, 3).

**Table 1:** General information comparison.

Group	*n*	Male/Female	Gestational Age (weeks)	Birth weight (g)
Fish oil group	41	20/21	31.67 ± 2.28	1,593.16 ± 159.32
Non-fish oil group	46	25/21	31.54 ± 2.05	1,587.25 ± 160.26
*χ^2^*/*t*	*–*	0.269	0.280	0.173
*P*	*–*	0.604	0.780	0.863

Data are presented as mean ± standard deviation or *n* (%). χ^2^ test for gender; independent *t*-test for gestational age and birth weight.

**Table 2:** Comparison of prenatal risk factors between the two groups.

Group	*n*	Maternal hypertension	Chorioamnionitis	Cesarean delivery	Intrauterine growth restriction	Antenatal Corticosteroids
Fish oil group	41	6 (14.63%)	4 (9.76%)	29 (70.73%)	5 (12.20%)	35 (85.37%)
Non-fish oil group	46	7 (15.22%)	5 (10.87%)	33 (71.74%)	6 (13.04%)	39 (84.78%)
*χ^2^*	*–*	0.006	0.000	0.011	0.014	0.006
*P*	*–*	0.939	1.000	0.917	0.905	0.939

Data presented as *n* (%). Group comparisons by χ^2^ test or Fisher's exact test when expected cell counts were ≤5.

**Table 3:** Comparison of neonatal risk factors between the two groups.

Group	*n*	Apgar score		Intubation at resuscitation	Surfactant administration	Invasive ventilation duration (days)
		1 min	5 min			
Fish oil group	41	6.24 ± 1.37	7.81 ± 0.89	12 (29.27%)	28 (68.29%)	8.53 ± 3.17
Non-fish oil group	46	6.08 ± 1.54	7.74 ± 1.02	15 (32.61%)	32 (69.57%)	9.06 ± 3.42
*χ^2^*	*–*	0.500	0.328	0.113	0.016	0.747
*P*	*–*	0.618	0.744	0.737	0.898	0.457
Group	*n*	FiO₂ at day 7 (%)	PDA	NEC (Bell's stage ≥2)	BPD	Sepsis
Fish oil group	41	32.14 ± 6.44	10 (24.39%)	3 (7.32%)	9 (21.95%)	7 (17.07%)
Non-fish oil group	46	33.54 ± 7.21	12 (26.09%)	4 (8.70%)	11 (23.91%)	8 (17.39%)
*χ^2^*	*–*	0.953	0.033	0.000	0.047	0.002
*P*	*–*	0.343	0.856	1.000	0.828	0.969

Data presented as mean ± SD or *n* (%). Group comparisons: independent *t*-test for continuous variables; Categorical variables analyzed by χ^2^ test or Fisher's exact test when expected cell counts were ≤5 test for categorical variables. PDA, patent ductus arteriosus; NEC, necrotizing enterocolitis; BPD, bronchopulmonary dysplasia.

### 3.2 Comparison of fatty acid profile levels between the two groups of patients

Table 4 reveals that there were no significant differences in the levels of DHA (1.72 ± 0.16 vs. 1.80 ± 0.13, *t* = 0.964, *P* = 0.338), EPA (2.33 ± 0.26 vs. 2.27 ± 0.25, t = 1.097, *P* = 0.276), and AA (12.06 ± 1.58 vs. 12.32 ± 1.42, *t* = 0.808, *P* = 0.421) between the two groups at the time of admission. However, the group receiving fish oil demonstrated significantly higher levels of DHA (2.37 ± 0.21 vs. 1.54 ± 0.13, *t* = 22.424, *P* < 0.001) compared to the group not receiving fish oil. Additionally, the fish oil group exhibited significantly lower levels of AA (8.99 ± 0.85 vs. 10.41 ± 1.69, *t* = 4.858, *P* < 0.001) compared to the non-fish oil group. These findings suggest that the inclusion of n-3 fatty acids in parenteral nutrition enhances DHA levels while reducing AA levels in premature infants.

**Table 4:** Comparison of fatty acid profile levels between the two groups of patients.

Group	*n*	DHA		EPA		AA	
		Day 1	Day 14	Day 1	Day 14	Day 1	Day 14
Fish oil group	41	1.77 ± 0.16	2.37 ± 0.21	2.33 ± 0.26	2.42 ± 0.27	12.06 ± 1.58	8.99 ± 0.85
Non-fish oil group	46	1.80 ± 0.13	1.54 ± 0.13	2.27 ± 0.25	2.49 ± 0.23	12.32 ± 1.42	10.41 ± 1.69
*t*	*–*	0.964	22.424	1.097	1.306	0.808	4.858
*P*	*–*	0.338	<0.001	0.276	0.195	0.421	<0.001

DHA, docosahexaenoic acid; EPA, eicosapentaenoic acid; AA, arachidonic acid. Values represent % of total fatty acids (mean ± SD). Group comparisons by independent *t*-test.

### 3.3 Comparison of time to recover birth weight, time to achieve full enteral feeding, and mechanical ventilation duration between the two groups during hospitalization

As shown in Table 5, there were no significant differences between the two groups in terms of time to recover birth weight (9.12 ± 0.98 vs. 8.98 ± 0.91, *t* = 0.691, *P* = 0.492) and duration of mechanical ventilation (14.41 ± 1.28 vs. 13.88 ± 1.25, *t* = 1.952, *P* = 0.054). However, the fish oil group had a significantly shorter time to achieve full enteral feeding (31.07 ± 3.18 vs. 40.42 ± 4.05, *t* = 11.874, *P* < 0.001) compared to the non-fish oil group. These findings suggest that the inclusion of n-3 fatty acids in enteral nutrition can promote intestinal function in patients.

**Table 5:** Comparison of time to recover birth weight, time to achieve full enteral feeding, and mechanical ventilation duration between the two groups during hospitalization (days).

Group	*n*	Time to recover birth weight (±SD)	Time to achieve full enteral feeding (±SD)	Mechanical ventilation duration (±SD)
Fish oil group	41	9.12 ± 0.98	31.07 ± 3.18	14.41 ± 1.28
Non-fish oil group	46	8.98 ± 0.91	40.42 ± 4.05	13.88 ± 1.25
*t*	*–*	0.691	11.874	1.952
*P*	*–*	0.492	<0.001	0.054

Data presented as mean ± SD (days). Group comparisons by independent *t*-test.

### 3.4 Comparison of hematological parameters between the two groups of patients

Table 6 demonstrates that there were no significant differences in platelet count, white blood cell count, red blood cell count, and hemoglobin level between the two groups on Day 1 (*t* = 0.015, 0.674, 0.649, 0.469; *P* = 0.988, 0.502, 0.518, 0.640) and Day 14 (*t* = 0.066, 1.580, 1.722, 1.235; *P* = 0.947, 0.118, 0.089, 0.220). These findings suggest that hematological parameters of preterm infants receiving different types of enteral nutrition were not significantly different, indicating that the choice of enteral nutrition strategy did not have an impact on the blood parameters.

**Table 6:** Comparison of hematological parameters between the two groups of patients (±SD).

Group	Platelets (10^9^/L)		White blood cells (10^9^/L)		Red blood cells (10^12^/L)		Hemoglobin (g/dl)	
	Day 1	Day 14	Day 1	Day 14	Day 1	Day 14	Day 1	Day 14
Fish oil group	192.49 ± 19.27	202.23 ± 20.34	13.28 ± 1.89	17.23 ± 1.06	3.98 ± 0.32	3.46 ± 0.34	15.78 ± 2.61	12.94 ± 3.17
Non-fish oil group	192.55 ± 19.10	202.52 ± 20.36	13.51 ± 1.26	16.85 ± 1.17	3.93 ± 0.39	3.34 ± 0.31	15.52 ± 2.55	12.11 ± 3.09
*t*	0.015	0.066	0.674	1.580	0.649	1.722	0.469	1.235
*P*	0.988	0.947	0.502	0.118	0.518	0.089	0.640	0.220

Data presented as mean ± SD. Group comparisons by independent *t*-test.

### 3.5 Comparison of liver function parameters between the two groups of patients

**Figure 1:**
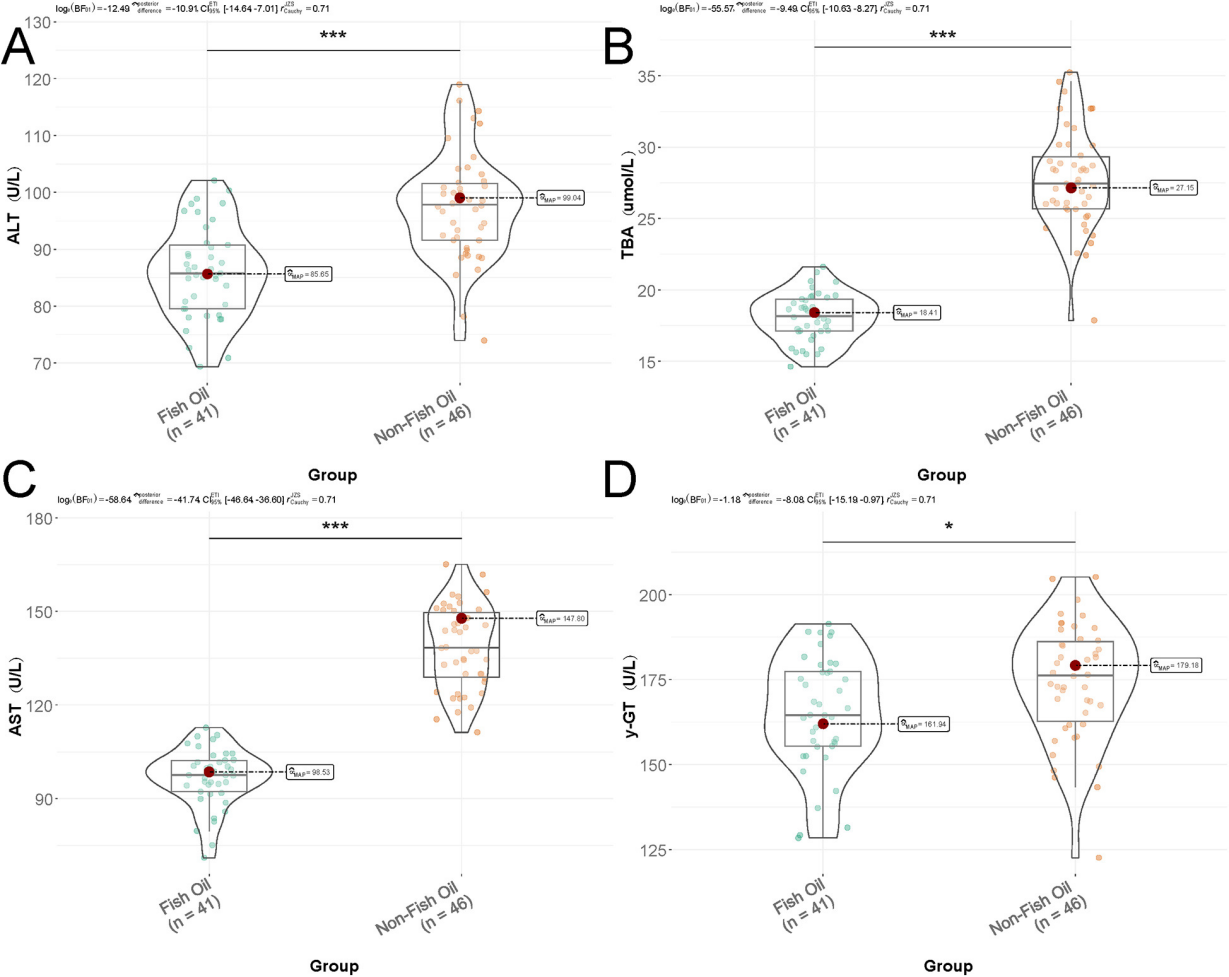
Comparison of liver function parameters between the Two groups. **(A)** ALT: Alanine aminotransferase (U/L); **(B)** TBA: total bile acid (μmol/L); **(C)** AST: Aspartate aminotransferase (U/L); **(D)** γ-GT: Gamma-glutamyl transferase (U/L). Data presented as mean ± SD. Group comparisons by independent *t*-test.

Figure 1 presents the results indicating significantly lower levels of ALT (86.07 ± 8.18 vs. 97.42 ± 9.25, *t* = 6.031, *P* < 0.001), TBA (18.12 ± 1.68 vs. 27.68 ± 3.45, *t* = 16.115, *P* < 0.001), AST (96.71 ± 9.28 vs. 138.68 ± 13.25, *t* = 16.914, *P* < 0.001), and γ-GT (165.15 ± 16.86 vs. 174.26 ± 17.15, *t* = 2.493, *P* = 0.015) in the Fish Oil Group compared to the Non-Fish Oil Group. These findings suggest that the inclusion of n-3-rich fish oil preparations resulted in superior improvements in liver function and demonstrated significant advantages in premature infants.

### 3.6 Comparison of ROP incidence in two groups

As shown in Table 7, there were no significant differences between the two groups in terms of overall ROP incidence and mild ROP incidence at 4 weeks post-admission (46.34% vs. 54.35%, 41.46% vs. 34.78%, *χ*^2^ = 0.556, 0.411, *P* = 0.456, 0.522) and 6 weeks post-admission (41.46% vs. 60.87%, 39.02% vs. 43.49%, *χ*^2^ = 3.270, 0.177, *P* = 0.071, 0.674). However, the administration of fish oil in the fourth (4.88% vs. 19.57%, *χ*^2^ = 4.069, *P* = 0.044) and sixth (2.44% vs. 17.39%, *χ*^2^ = 5.226, *P* = 0.022) weeks resulted in a significantly lower incidence of severe ROP compared to the group not receiving fish oil. These findings indicate that the provision of n-3-rich fish oil preparations has a positive effect in reducing the occurrence of severe ROP in premature infants.

**Table 7:** Comparison of ROP incidence and degree between the two groups [*n* (%)].

Group	*n*	Overall ROP		Mild ROP		Severe ROP	
		4 weeks	6 weeks	4 weeks	6 weeks	4 weeks	6 weeks
Fish oil group	41	19 (46.34%)	17 (41.46%)	17 (41.46%)	16 (39.02%)	2 (4.88%)	1 (2.44%)
Non-fish oil group	46	25 (54.35%)	28 (60.87%)	16 (34.78%)	20 (43.49%)	9 (19.57%)	8 (17.39%)
*χ^2^*	*–*	0.556	3.270	0.411	0.177	4.069	5.226
*P*	*–*	0.456	0.071	0.522	0.674	0.044	0.022

Data presented as *n* (%). Group comparisons: χ^2^ test for Overall ROP and Mild ROP; Severe ROP at 4 weeks: χ^2^ test (expected counts >5); Severe ROP at 6 weeks: Fisher's exact test* (due to low expected frequency in fish oil group); ROP severity classified per ICROP criteria. ROP severity classified per ICROP criteria.

### 3.7 Comparison of ROP surgical rates between the two groups

Table 8 demonstrates that the fish oil group had no incidences requiring surgical treatment, while the non-fish oil group had five cases of surgery, including four treated with laser therapy and one undergoing vitreoretinal surgery. The surgical rate in the fish oil group (0.00% vs. 15.22%, *χ*^2^ = 4.728, *P* = 0.030) was significantly lower compared to the non-fish oil group. These findings indicate that the administration of n-3-rich fish oil preparations has a positive effect in reducing the rate of surgical interventions for ROP in premature infants.

**Table 8:** Comparison of ROP surgical rates between the Two groups [*n* (%)].

Group	*n*	Surgery	Surgical rate
Fish oil group	41	0 (0.00)	0 (0.00)
Non-fish oil group	46	5 (15.22)	5 (15.22)
*χ^2^*	*–*	*–*	4.728
*P*	*–*	*–*	0.030

Surgical interventions included laser therapy and vitrectomy. Group comparison by Fisher's exact test.

## 4 Discussion

The vasculogenesis of the retina begins around the 14th week of gestation and continues until birth. Premature infants, who have not completed all the critical stages of retinal development, often have immature retinas with avascular areas. As a result, these infants require external support to promote retinal development and complete vascularization, which is crucial in preventing various ocular diseases and visual developmental disorders (14, 15). Pathological neovascularization and fibroproliferation occur in the immature retina of premature infants during this developmental process, forming the pathological basis of ROP. Prematurity, low birth weight, and oxygen therapy are recognized as the primary risk factors for ROP (16). Therefore, facilitating healthy postnatal retinal development and reducing pathological neovascularization are key in preventing ROP.

After birth, premature infants require adequate nutrition to support their growth, development, and organ function, including the retina. The avascular area of the retina increases the risk of developing ROP, especially for infants with smaller gestational age and lower birth weight (17). Sufficient nutritional support plays a vital role in providing the necessary nutrients and energy for retinal development. The central nervous system cells and retinal vascular epithelial cells of premature infants continue to develop after birth, requiring ongoing nutritional support for cell division and growth. Insufficient nutrition during this critical period may halt retinal vascular epithelial cell division, leading to impaired retinal maturation and eventual progression to ROP. Additionally, DHA is essential for retinal development, as it serves as a building block for the synthesis of cone and rod cells (18, 19). Research has shown that fish oil emulsions rich in n-3 fatty acids have a preventive effect against severe ROP in premature infants (20). A prospective observational study also revealed a significantly reduced incidence of ROP with increased serum DHA levels, suggesting a potential association between DHA levels and ROP occurrence (21). However, other study, including a retrospective comparative study, did not find significant differences in ROP occurrence between the n-3 fatty acid emulsion group and control group (22). These varying results may be attributed to differences in the duration of fish oil administration in respective studies (23).

This study demonstrated significantly higher levels of DHA in the fish oil group compared to the non-fish oil group. Early DHA supplementation in premature infants was found to be associated with a lower risk of severe ROP (24). While DHA is usually provided to the fetus during pregnancy through the placenta, premature infants often start breastfeeding later than full-term infants. Additionally, maternal milk supply in premature infants may be inadequate in terms of DHA content, necessitating early use of PN to meet their growth and development needs (25, 26). Therefore, early provision of fish oil emulsion rich in n-3 fatty acids in premature infants' PN can fulfill DHA requirements and contribute to their growth and development. The study also observed a significant decrease in the ratio of AA in the fish oil group compared to the non-fish oil group after fat emulsion supplementation. AA derivatives contribute to vascular injury through platelet activation and TRPM2 channel modulation, while higher serum AA levels are associated with a preventive effect against ROP by maintaining metabolic balance and reducing pathological angiogenesis (27, 28). While elevated DHA levels were expected with fish oil emulsion, the significant reduction in AA and the resultant shift in the DHA/AA ratio likely underpin the observed reduction in severe ROP and surgical need, suggesting a synergistic biochemical mechanism beyond isolated DHA supplementation. AA is essential for DHA synthesis in the human body (29). Lower AA levels and higher DHA levels indicate increased DHA synthesis, which has a protective effect on the retina of premature infants. Other studies have also indicated a negative correlation between serum AA levels and ROP incidence (21). Fish oil emulsion has been shown to reduce AA concentration in a meta-analysis (30). Additionally, increasing DHA concentration and low AA concentration were associated with a lower incidence of ROP (31).

The study also observed significantly lower levels of liver enzymes (ALT, TBA, AST, γ-GT) in the fish oil group compared to the non-fish oil group. Impaired liver function is commonly observed in premature infants and can lead to complications such as jaundice, abnormal liver function, and cell damage. Fish oil emulsion, as a nutritional supplement, can improve liver function in premature infants through various mechanisms. Firstly, fish oil emulsion is rich in polyunsaturated fatty acids like EPA and DHA, which have a protective effect on liver function by reducing oxidative stress and inflammatory reactions (32). Secondly, the antioxidant compounds present in fish oil emulsion inhibit oxidative stress reactions, reducing the production of free radicals and protecting liver cells from damage. Fish oil emulsion's antioxidants alleviate oxidative stress-induced impaired liver function in premature infants (33, 34). Lastly, fish oil emulsion contains linoleic acid, an essential fatty acid crucial for the development of liver function in premature infants. Linoleic acid contributes to the synthesis and stability of liver cell membranes, promoting their normal functionality. The presence of linoleic acid in fish oil emulsion provides additional supply, thereby improving liver function in premature infants (35).

This study found that the fish oil group achieved full enteral feeding in a significantly shorter amount of time compared to the non-fish oil group. However, the fish oil group had a higher incidence of severe ROP at weeks 4 and 6, while the surgical rate for ROP was significantly lower in the fish oil group compared to the non-fish oil group. Several factors explain these observations. Firstly, fish oil emulsion provides essential fatty acids, including omega-3 polyunsaturated fatty acids (e.g., EPA and DHA), which are crucial for retinal development. These fatty acids promote normal growth and development of retinal cells and maintain the normal structure and function of the retina (27). Secondly, omega-3 polyunsaturated fatty acids have anti-inflammatory properties that regulate inflammatory responses and reduce retinal damage caused by inflammation. Fish oil emulsion's anti-inflammatory effects help alleviate the severity of retinopathy by suppressing inflammatory responses and reducing the release of inflammatory mediators. Thirdly, the antioxidant properties of omega-3 polyunsaturated fatty acids enable them to scavenge free radicals and reduce oxidative damage. ROP is associated with oxidative damage, and the antioxidant effects of fish oil emulsion protect retinal cells from oxidative damage, mitigating the severity of retinopathy. Lastly, omega-3 polyunsaturated fatty acids in fish oil emulsion promote neuroprotection and repair. ROP often involves neuronal damage and neurodegenerative changes, and fish oil emulsion promotes the growth, development, and protection of nerve cells, thereby lessening the extent of retinopathy.

This study has several limitations. First, its retrospective design and non-randomized allocation introduce potential selection bias and restrict control over confounding variables (e.g., antenatal inflammation, growth restriction, resuscitation details, and oxygen protocols). Although prenatal/neonatal factors were comparable, unmeasured confounders like breastmilk composition or enteral formula differences could influence outcomes. Second, the small sample size (*n* = 87) and limited severe ROP cases (*n* = 5 surgeries) reduce statistical power for subgroup analyses. Third, we did not account for anti-VEGF therapies or emerging pharmacological agents (e.g., propranolol), which may alter ROP progression and surgical needs. Finally, long-term neurodevelopmental and visual outcomes were not assessed. Future prospective, randomized trials with larger cohorts should standardize nutritional protocols, document enteral nutrition details, track anti-VEGF usage, and include longitudinal follow-up to evaluate sustained effects of lipid emulsions on ROP severity and neurodevelopment.

In conclusion, early use of n-3 fatty acid-enriched lipid emulsion in premature infants has a preventive effect on severe ROP by maintaining higher serum DHA levels and lower AA levels. This intervention significantly reduces the time required to achieve full enteral feeding and the surgical rate for ROP.

## Data Availability

The raw data supporting the conclusions of this article will be made available by the authors, without undue reservation.
